# PAPP5 Is Involved in the Tetrapyrrole Mediated Plastid Signalling during Chloroplast Development

**DOI:** 10.1371/journal.pone.0060305

**Published:** 2013-03-29

**Authors:** Juan de Dios Barajas-López, Dmitry Kremnev, Jehad Shaikhali, Aurora Piñas-Fernández, Åsa Strand

**Affiliations:** Umeå Plant Science Centre, Department of Plant Physiology, Umeå University, Umeå, Sweden; University of Delhi South Campus, India

## Abstract

The initiation of chloroplast development in the light is dependent on nuclear encoded components. The nuclear genes encoding key components in the photosynthetic machinery are regulated by signals originating in the plastids. These plastid signals play an essential role in the regulation of photosynthesis associated nuclear genes (*PhANGs*) when proplastids develop into chloroplasts. One of the plastid signals is linked to the tetrapyrrole biosynthesis and accumulation of the intermediates the Mg-ProtoIX and its methyl ester Mg-ProtoIX-ME. Phytochrome-Associated Protein Phosphatase 5 (PAPP5) was isolated in a previous study as a putative Mg-ProtoIX interacting protein. In order to elucidate if there is a biological link between PAPP5 and the tetrapyrrole mediated signal we generated double mutants between the Arabidopsis *papp5* and the *crd* mutants. The *crd* mutant over-accumulates Mg-ProtoIX and Mg-ProtoIX-ME and the tetrapyrrole accumulation triggers retrograde signalling. The *crd* mutant exhibits repression of *PhANG* expression, altered chloroplast morphology and a pale phenotype. However, in the *papp5crd* double mutant, the *crd* phenotype is restored and *papp5crd* accumulated wild type levels of chlorophyll, developed proper chloroplasts and showed normal induction of *PhANG* expression in response to light. Tetrapyrrole feeding experiments showed that PAPP5 is required to respond correctly to accumulation of tetrapyrroles in the cell and that PAPP5 is most likely a component in the plastid signalling pathway down stream of the tetrapyrrole Mg-ProtoIX/Mg-ProtoIX-ME. Inhibition of phosphatase activity phenocopied the *papp5crd* phenotype in the *crd* single mutant demonstrating that PAPP5 phosphatase activity is essential to mediate the retrograde signal and to suppress *PhANG* expression in the *crd* mutant. Thus, our results suggest that PAPP5 receives an inbalance in the tetrapyrrole biosynthesis through the accumulation of Mg-ProtoIX and acts as a negative regulator of *PhANG* expression during chloroplast biogenesis and development.

## Introduction

The chloroplasts house the photosynthetic light reactions where sunlight is converted into chemical energy in the form of NADPH and to an electrochemical gradient over the thylakoid membrane that is subsequently used to synthesize ATP. Plastids are also the location of a number of vital metabolic pathways, including primary carbon metabolism and the biosynthesis of fatty acids, amino acids and tetrapyrroles. Plastids exhibit a very clear developmental program where all plastids are derived from proplastids present in meristematic cells either direct or via the dark-grown intermediate form known as etioplasts. The etioplasts contain a characteristic lattice-like membrane structure known as the prolamellar body. The prolamellar body also contains the precursor of chlorophyll, protochlorophyllide, bound to its reducing enzyme protochlorophyllide oxidoreductase (POR). Following exposure to light the prolamellar body forms the thylakoid membrane and POR is activated to convert protochlorophyllide into chlorophyllide a, which is subsequently converted into chlorophyll a and b [1]. Plants can detect almost all wavelengths of light using three major classes of photoreceptors: the red/far-red light absorbing phytochromes, the blue/UV-A light absorbing cryptochromes and phototropins, and the UV-B sensing UV-B receptors [2]. These photoreceptors perceive light signals and initiate intracellular signalling pathways involving proteolytic degradation of signalling components and large reorganization of the transcriptional program to modulate plant growth and development [2]. When dark grown seedlings are exposed to light as much as one-third of the nuclear encoded genes show transcription changes [3] and among the genes dramatically up-regulated in the light are genes encoding chloroplast-targeted proteins. Thus, it is clear that the initiation of chloroplast development in the light is dependent on nuclear encoded components through so called anterograde mechanisms [4]. Although the control of chloroplast development appears to be overwhelmingly under nuclear control there are several reports demonstrating that nuclear genes encoding key components in the photosynthetic machinery are regulated by signals originating in the plastids, so called retrograde mechanisms [5], [6], [7].

During chloroplast development a tight stoichiometric assembly of nuclear-encoded and plastid-encoded proteins together with chlorophylls and carotenoids is essential. To achieve this there must be a communication between the chloroplast and the nucleus through retrograde signalling. The first evidence of the existence of a “plastid signal” came from studies of mutants with morphologically aberrant plastids. These include mutants with defective plastid protein synthesis such as the plastid ribosome-deficient *albostrians* barley mutant and the *Brassica napus al* mutant [8], [9], [10]. These mutants demonstrated reduced expression of nuclear-encoded plastid components suggesting that a plastid signal was emitted to repress the nuclear encoded photosynthesis genes [10]. We now know that several different plastid processes produce signals that regulate specific sets of genes or regulons and several molecular candidates for plastid signals have been described [11]. Plastid signals are essential to the plant both during the initial developmental stages (biogenic control) and in adult stage to face changes in the environment (operational control) [12].

One of the plastids signals described to regulate the expression of photosynthesis associated nuclear genes (*PhANGs*) is linked to the tetrapyrrole biosynthesis and accumulation of the intermediates the Mg-ProtoIX and its methyl ester Mg-ProtoIX-ME [13], [14], [15], [16], [17], [18]. Mg-ProtoIX and Mg-ProtoIX-ME have been shown to accumulate when plants and algae are exposed to factors that give rise to oxidative stress such as exposure to low temperatures and inhibitors of photosynthetic electron transport [18], [19], [20], [21]. The reason for the accumulation of these particular intermediates could be explained by the fact that the aerobic cyclase reaction was shown, both in Arabidopsis and cucumber, to be extremely sensitive to oxidative stress [20], [21]. Thus, impaired flux through chlorophyll biosynthesis and the accumulation of Mg-ProtoIX/Mg-ProtoIX-ME is an indicator of changes in the environment and results in changes in *PhANG* expression. Whether accumulation of Mg-ProtoIX is itself an important part of the tetrapyrrole-mediated signal remains unclear [22], [23]. In order to investigate whether Mg-ProtoIX itself is an important part of the tetrapyrrole-mediated signal, cytosolic ligands of Mg-ProtoIX were isolated from a proteomic study [17] and the Phytochrome-Associated Protein Phosphatase 5 (PAPP5) was isolated as a one of the putative Mg-ProtoIX interacting proteins [17]. PAPP5 is a type 5 serine/threonine protein phosphatase that dephosphorylates biologically active Pfr phytochromes and enhances the phytochrome-mediated photoresponses. Depending on the specific serine residues dephosphorylated by PAPP5, phytochrome stability and affinity for a downstream signal transducer, NDPK2, were enhanced [24]. Chloroplast biogenesis and development is triggered by exposure to light [1], [25] and possibly PAPP5 is the suggested link between plastid and light signalling networks [26], [27].

In order to elucidate if there is a biological link between PAPP5 and the tetrapyrrole mediated signal we prepared double mutants between the *papp5* mutant and the *chl27/crd* Arabidopsis mutant. *CHL27/CRD* is a nuclear gene encoding one of the potential subunits of the aerobic Mg-protoporphyrin monomethyl ester cyclase complex and the *crd* mutant over-accumulates Mg-ProtoIX and Mg-ProtoIX-ME and the tetrapyrrole accumulation affects the retrograde signalling in the *crd* mutant [28]. Surprisingly, introducing the *papp5* mutation into the *crd* background reverts the pale phenotype and restores normal chloroplast development and light stimulated induction of *PhANG* expression in *crd*. Our results suggest that PAPP5 is a component in the tetrapyrrole-mediated plastid signalling pathway and genetic, physiological and biochemical data supporting this model will be discussed.

## Materials and Methods

### Plant material and growth conditions

The different *Arabidopsis thaliana* lines used in this study were Col-0 wild type, *crd*/*chl27* (Salk_009052) and *papp5* (Salk_021153) (Figure S1). T-DNA insertion lines were obtained from NASC stock centre. For aseptic growth, seeds were surface-sterilized with 95% Ethanol and 0.5% Triton X100 (v/v), washed tree times with 95% ethanol, stratified at 4°C for 2 days in the dark and spread on 1% sucrose MS agar plates complemented with vitamins (Duchefa). Seed germination was induced by exposing plates to 100 µmol photons m^−2^ s^−1^ light, 22°C for 12 hours. Plates were transferred to darkness for 3 days and then shifted to growth cabinet in continuous light during 24 hours (100 µmol photons m^−2^ s^−1^, 22°C). Mature plants were grown on soil for 6 weeks (100 µmol photons m^−2^ s^−1^, 9 hr light/15 hr darkness, 22°C).

### Morphological and physiological analysis

For chlorophyll content analysis, 6-weeks-old plants grown on soil at 100 µmol photons m^−2^ s^−1^, 9 hr light/15 hr darkness, 22°C. Samples were ground with liquid nitrogen and 1 mL of buffered acetone (80% acetone, 25 mM HEPES pH 7.5) was added to 100 mg of material and incubated over night at 4°C. Chlorophyll content was determined according to Porra [29]. For TEM, 6-weeks-old plants grown on soil and seedlings grown on MS 1× agar plates supplemented with 1% sucrose were prepared, embedded and cut according to Keskitalo *et al*. [30]. Flowering time was determined by counting the number of leaves and days when floral buds were visible at the centre of the rosette.

### cDNA synthesis and Real-Time PCR analysis

Total RNA was isolated using Total RNA Miniprep kit (EZNA) according to the manufacturer's instructions. Total RNA concentration was determined with a Nanodrop ND-100 spectrophotometer. cDNA was synthesized from 0.5 µg of total RNA using the iScript cDNA synthesis kit (Bio-Rad) according to the manufacturer's instructions. 2 µl of 10-fold diluted cDNA was used in 10 µl iQ SYBR Green Supermix reaction (BioRad). All reactions were performed in triplicates. The different sets of primers used are described in Table S1. RT-PCR was run in a CFX96 real time system (BioRad) and analyzed using LinRegPCR [31], [32].

### ALA/Mg-ProtoIX feeding experiment and tetrapyrrole quantification

Seedlings were grown on MS agar plates supplemented with 1% sucrose for 2 weeks under long day conditions (100 µmol photons m^−2^ s^−1^, 15 hr light/9 hr darkness, 22°C). Liquid medium containing 50 µM of Mg-ProtoIX (Frontier Scientific Inc.) in 1× MS (Murashige and Skoog medium) supplemented with 1% sucrose was spread on the plates before the dark period and samples were collected the next day in the middle of the light period. Mock plates were treated with same medium but without Mg-ProtoIX. For ALA feeding treatment, 2 weeks old seedlings grown in 1× MS plates 1% sucrose under long day conditions (100 µmol photons m^−2^ s^−1^, 15 hr light/9 hr darkness, 22°C) were incubated overnight in darkness in 10 mM potassium phosphate, pH 7.5, 5 mM MgCl_2_, 10 mM 5-aminolevulinic acid (SIGMA-Aldrich) and transferred back to light conditions for one hour before harvesting. Mock samples were incubated in the same phosphate buffer but without 5-aminolevulinic acid. To determine tetrapyrrole content, samples were homogenized in liquid nitrogen and extracted with acetone/1 M NH_4_OH (99∶1; v/v) under dim green light at 4°C. Samples were continuously shaken during 40 min and spun for 10 min at 14000 rpm, 4°C. The supernatant was transferred to a fresh microcentrifuge tube and extracted twice with equal volume of hexane. Mg-ProtoIX/Mg-ProtoIX-ME fluorescence emission spectra from 460 to 720 nm of the acetone fraction was recorded at room temperature by using the excitation wavelength 416 nm. Final data analysis was made using Igor Pro software (Ver. 6.22A).

### Okadaic acid treatment

Seedlings were grown on filter paper soaked with MS medium and 1% sucrose. The plates were transferred to 100 µmol photons m^−2^ s^−1^ constant light and 22°C for 12 hours to induce germination. Plates were transferred to darkness for 3 days and then shifted from darkness to light (24 hours at 100 µmol photons m^−2^ s^−1^, 22°C). During the light incubation, the seedlings were treated with 10 nM okadaic acid (SIGMA) diluted in 1× MS medium supplemented with 1% sucrose medium. For the mock treatment, okadaic acid was excluded.

### Isolation of thylakoid protein complexes and Blue Native PAGE

5 weeks old Arabidopsis plants grown in short day were used to isolate chloroplasts in a two-step 50%–25% percoll gradient described previously [33]. Thylakoid membrane purification was done according to Hall *et al*. 2011 [34]. Protein complexes from isolated thylakoids were solubilized in BN-solubilization buffer (30 mM HEPES, pH 7.4; 150 mM potassium acetate; 10% glycerole, 4% digitonin (SIGMA); 1% β-Dodecylmaltoside (SIGMA) for 40 min, 4°C. Unsolubilized material was removed by centrifugation at 14000 g, 10 min. 35 µg of protein was loaded on 4–12% Bis-Tris Gel (NuPAGE® Novex 1.0 mm, Invitrogen).

### Binding assay

Immunoprecipitation of native PAPP5 complex was performed using 4-weeks old *Petita havana* tobacco plants transfected with 35S:PAPP5-cMyc expression construct. Tobacco leaves were collected and grinded in liquid nitrogen. Proteins from the leaf tissue were extracted in 1000 µL immunoprecipitation buffer (25 mM Tris- HCl, pH 7.8, 75 mM NaCl, 10 mM MgCl_2_, 2 mM dithiothreitol (DTT), 5 mM EGTA, 0.2% Triton X-100, 10% glycerol, 0.2 mM PMSF) for 60 min, 4°C. Extracts were incubated with 5 mg of anti-cMYC monoclonal antibody (Bio-Site) bound to protein G coated magnetic beads (Dynabeads Protein G Immunoprecipitation, Invitrogen) for 1 h at 4°C. All the washing steps were performed according to the manufacturer's recommendations. The immunoprecipitated protein complex was resuspended in 200 µL of PBS buffer (pH 7.4) and incubated for 1 h with 2.5 µM Mg-ProtoIX at 4°C. Beads were washed with 1 ml PBS three times and proteins were eluted with Native elution buffer. Mg-ProtoIX bound to the PAPP5 complex was quantified by spectrofluorometry with the excitation wavelength at 416 nm. Sample of Mg-ProtoIX incubated with immunoprecipitated proteins from tobacco plants transfected with pGWB16 control plasmid was used as the negative control.

## Results

### The *papp5* mutation reverts the pale *crd* phenotype in *papp5crd* double mutant

**Figure 1:**
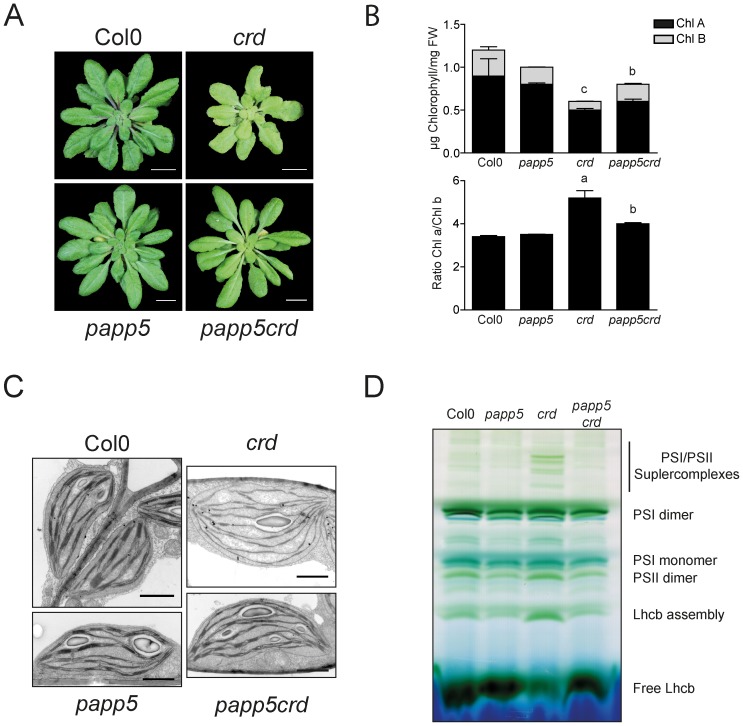
The *papp5* mutation rescues the *crd* phenotype. Plants were grown on soil under short day (SD) conditions (9 hours light/15 hours darkness) to characterize wild type, *crd*, *papp5* and *papp5crd*. A) Representative images from 6-week-old plants. Scale bars represent 1 cm. B) Chlorophyll a and b content and chlorophyll a/b ratio in 6-week-old plants. Significant differences relative to Col0 (*crd*) and to *crd* (*papp5crd*) according to *t-test* (a, P<0.001; b, P<0.005; c, P<0,01) are shown. C) Representative electron microscopy images of chloroplasts from Col0, *crd*, *papp5* and *papp5crd* from 6-week-old plants grown under SD conditions. Scale bar is 1 µm. D) Blue native PAGE profile of thylakoid membrane protein complexes isolated from Col0, *papp5*, *crd* and *papp5crd* plants. Each well was loaded with the 35 µg protein.

We used a T-DNA insertion mutant (*crd*) of the *CHL27/CRD* gene encoding a potential subunit of the cyclase enzyme complex involved in chlorophyll biosynthesis downstream of Mg-ProtoIX [28], [35]. The *crd* mutant has a pale phenotype and reduced growth (Figure 1A–B, Figure S2) [28], [35], [36]. The strong reduction in chlorophyll b content compared to chlorophyll a results in a higher chlorophyll a/b ratio in *crd* compared to wild type (Figure 1B). The T-DNA insertion line for PAPP5, *papp5* demonstrated wild type phenotype under our growth conditions. In the *papp5crd* double mutant the pale phenotype observed in the *crd* single mutant is lost and the chlorophyll content is recovered to wild type levels (Figure 1A–B) together with the recovery of the morphology and the flowering time (Figure S3).

In addition to the pale phenotype, the chloroplast structure in the *crd* mutant is different compared to wild type and *papp5*. The *crd* chloroplasts are poorly developed containing only stroma-exposed thylakoids and abnormal grana thylakoids (Figure 1C) [28]. Coinciding with the defect in chloroplast structure is the difference in the arrangements of the photosynthetic complexes in the *crd* mutant compared to wild type and the *papp5* mutant (Figure 1D). Similarly to what was observed for chlorophyll content (Figure 1A–B), the *papp5crd* double mutant reverts the chloroplast phenotype observed in the *crd* single mutant (Figure 1C–D). Thus, in contrast to the *crd* single mutant the *papp5crd* double mutant displays normal growth and chlorophyll content, fully developed chloroplast with proper thylakoid membranes and correctly assembled photosynthetic complexes (Figure 1A–D).

We hypothesized that the obtained phenotype in the *papp5crd* double mutant could be linked to a defective PHYA/PHYB signalling pathway. However, the *phyBcrd* double mutant maintained the pale phenotype of *crd* compared to wild type (Figure S2A–B). These results suggest that impairment in the *phyB* mediated light-signalling pathway is not involved in the restoration of *papp5crd* double mutant phenotype. We also tested the possibility that introducing the *papp5* mutation would revert the phenotype of any pale mutant unrelated to the accumulation of Mg-ProtoIX by generating the *papp5gun5* double mutant. The *gun5* mutant has a pale phenotype but it does not accumulate Mg-ProtoIX. This pale phenotype was maintained also in the *papp5gun5* double mutant (Figure S2C).

### The *papp5crd* double mutant demonstrates normal chloroplast development

**Figure 2:**
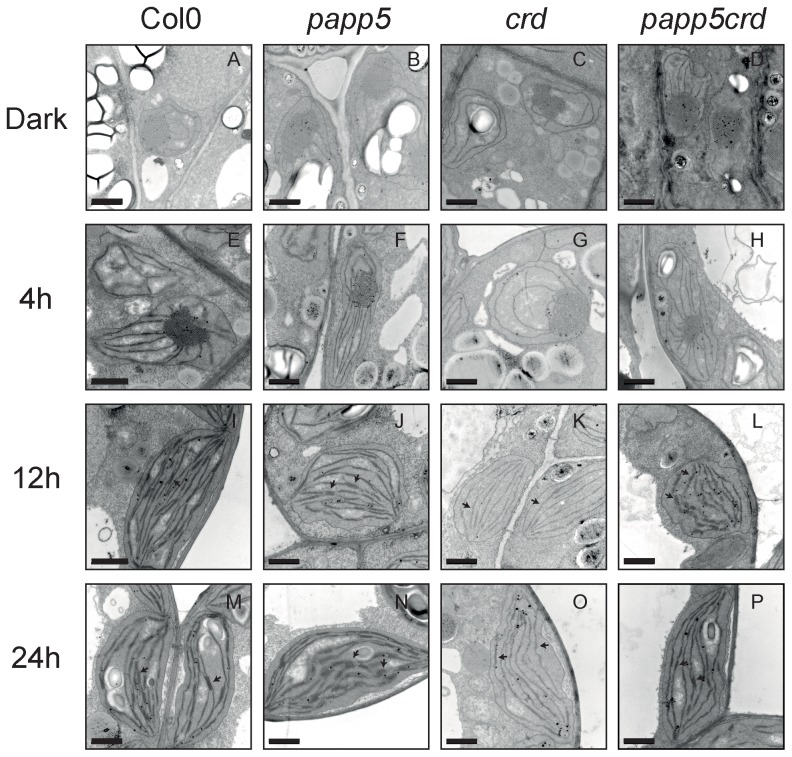
Chloroplast development during de-etiolation. Sequential electron microscopy images from Col0 (A, E, I and M), *papp5* (B, F, J and N), *crd* (C, G, K and O) and *papp5crd* (D, H, L and P) during the first 24 hours of illumination (100 µmol photons m^−2^ sec^−1^). Samples were collected 4 h, 8 h, 12 h and 24 h following transfer to light and compared to the dark sample. Arrows indicate examples of grana structures. Scale bar = 1 µm.

Depletion of PAPP5 restored the *crd* mutant phenotype in mature plants. To investigate if PAPP5 plays a role during chloroplast development and seedling establishment we analyzed the chloroplast morphology at different time points during the first 24 hours of seedling development in the light. In the dark grown samples, we observed no differences in either the prolamelar bodies (PLB) or the plastid structures between wild type and any of the mutants (Figure 2A–D). In wild type the first signs of chloroplast development was detected after 4 hours of illumination when the PLBs start to disappear and the first thylakoid membranes were formed (Figure 2E). A similar development of the chloroplasts was observed in the *papp5* mutant but in *papp5* it was also possible to detect formation of grana membranes already after 4 hours of light exposure (Figure 2F). In contrast, chloroplast development is significantly delayed in *crd* (Figure 2G). However, in the *papp5crd* double mutant the chloroplasts develop normally (Figure 2H). After 12 hours of illumination, the chloroplasts in wild type, *papp5* and *papp5crd* are already fully developed and the basic internal thylakoid membranes and grana stacks are formed. The chloroplasts in *crd* display less evolved thylakoid membranes that lack grana structures (Figure 2I–L). The same observation was made after 24 hours of illumination where the *crd* seedlings showed minimal thylakoid membrane structures without grana complexes (Figure 2O). However, this feature was restored in the *papp5crd* double mutant. These studies confirm that the changes that restore the *crd* phenotype in *papp5crd* start during the de-etiolation process.

**Figure 3:**
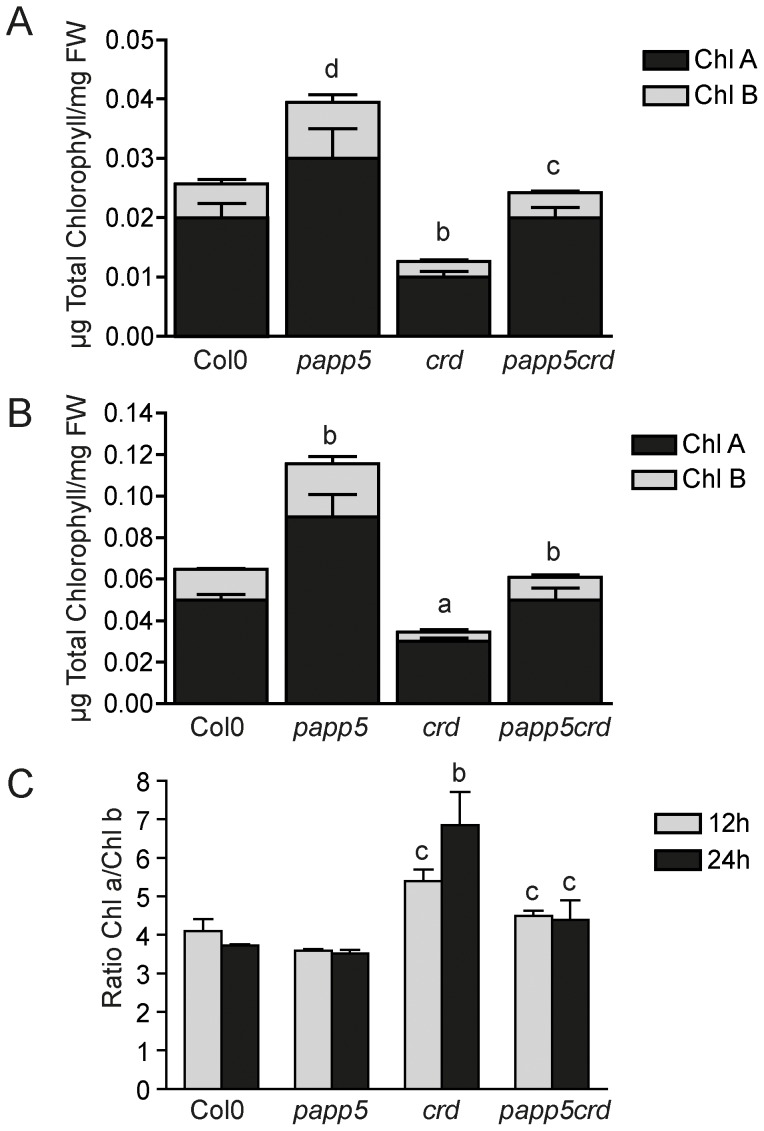
Chlorophyll determination during de-etiolation. Chlorophyll content in Col0, *papp5*, *crd* and *papp5crd* seedlings following A) 12 h and B) 24 h of illumination. C) Chlorophyll a/b ratio in the different lines during 12 h and 24 h of illumination. Significant differences relative to Col0 (*crd*) and to *crd* (*papp5crd*) according to *t-test* (a, P<0.001; b, P<0.005; c, P<0.01; d, P<0.05) are shown.

Similar changes were observed when we analyzed the chlorophyll content in the different genotypes during the first hours of seedlings development upon illumination. The *crd* accumulated significantly less chlorophyll compared to wild type following both 12 and 24 hours illumination (Figure 3A–B). In addition, the chlorophyll a/b ratio was elevated in the *crd* seedlings similarly to what was observed in the mature plants (Figure 3C and 1B). However, *papp5crd* double mutant demonstrated similar chlorophyll a and b levels and chlorophyll a/b ratio to wild type (Figure 3A–C). Interestingly, the *papp5* mutant demonstrated higher levels of chlorophyll following both 12 and 24 hours illumination suggesting that PAPP5 acts as a negative regulator of chlorophyll biosynthesis during the early light response (Figure 3A–B).

### Normal induction of *PhANG* expression in response to light is obtained in the *papp5crd* double mutant

**Figure 4:**
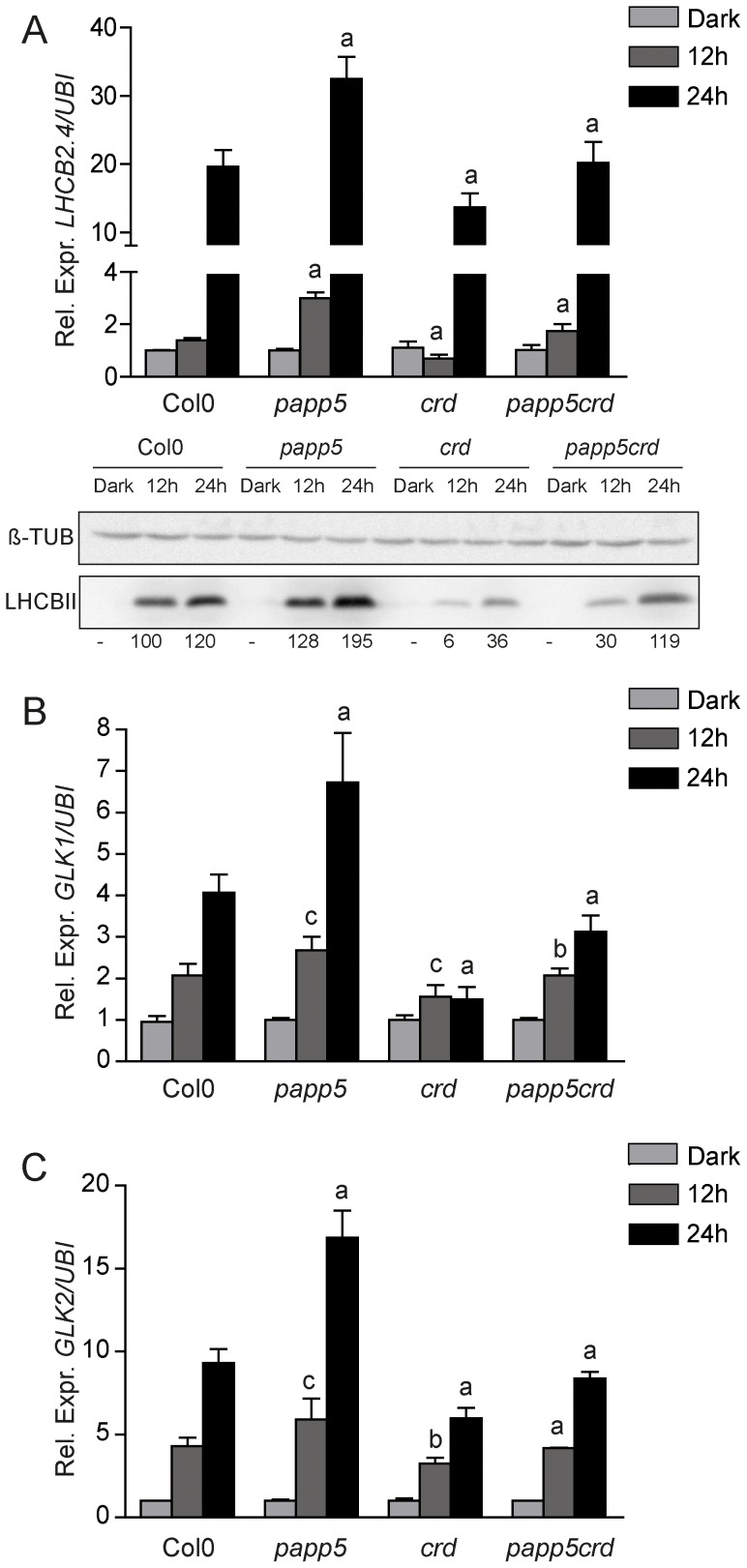
*PhANG* expression during chloroplast development. Relative expression levels of A) *LHCB2.4* (At3g27690) complemented with Western blot analysis of LHCBII protein and β-Tubulin as protein loading control, B) *GLK1* (At2g20570) and C) *GLK2* (At5g44190) in seedlings grown for three days in dark and exposed to 12 h and 24 h of illumination. Expression levels were compared to the respective dark control for each genotype and relative expression was calculated using Ubiquitin-protein ligase (At4g36800) as a reference gene. Data represents the mean (± SD) from three independent biological replicates. Significant differences relative to Col0 (*crd* and *papp5*) and to *crd* (*papp5crd*) were calculated according to *t-test* (a, P≤0.001; b, P≤0.005; c, P≤0.01). The bands were quantified using ImageJ software and the relative band intensities were obtained and related to Col0 12 h samples.

The different physiological features observed in *papp5crd* compared to the *crd* single mutant encouraged us to investigate expression of genes encoding components essential for chloroplast development. We analysed expression of the *PhANGs*, *LHCB2.4*, *GLK1* and *GLK2* during the first 12 and 24 hours of illumination. *LHCB2.4* is a nuclear gene encoding a chloroplast LHCII antenna apoprotein that is expressed during the light-induced chloroplast development. As expected, *LHCB2.4* expression increased following exposure to light compared to the dark samples in wild type (Figure 4A). In *papp5*, the *LHCB2.4* transcript levels increased more strongly following 12 and 24 hours light exposure compared to wild type. Also when expression levels were compared to wild type at each time point *PhANG* expression was higher in the *papp5* mutant (Figure S4). This coincides with the increased accumulation of chlorophyll that was also observed in the *papp5* mutant compared to wild type (Figure 3). In contrast, the *crd* mutant demonstrated impaired induction of *LHCB2.4* expression compared to wild type and *papp5*. However, the *papp5* mutation restores the suppressed *PhANG* expression in *crd* and the *papp5crd* double mutant showed significantly stronger induction of *LHCB2.4* expression compared to the *crd* single mutant (Figure 4A). The expression data was supported by the LHCB2.4 protein levels in the different genotypes as demonstrated by Western blot (Figure 4A).

The GLK1 and GLK2 are transcription factors required for chlorophyll biosynthesis and *PhANG* expression during chloroplast development [25], [37]. Similarly to the expression of *LHCB2.4*, expression of *GLK1/2* is induced by exposure to light in wild type (Figure 4B–C). Again, a stronger induction of *GLK1* and *GLK2* expression in response to light was observed in *papp5* compared to wild type whereas in *crd* expression of *GLK1* did not change significantly during the first 24 hours of light compared to the dark sample. Also the induction of *GLK2* expression was significantly suppressed in *crd* compared to what was observed in wild type (Figure 4B–C). The suppressed *PhANG* expression in *crd* coincides with the observed arrest of chloroplast development in the mutant (Figure 2). Finally, the *papp5crd* seedlings displayed *GLK1* and *GLK2* expression similar to wild type (Figure 4B–C). Also when expression levels were compared to *crd* at each time point *PhANG* expression was restored in the *papp5crd* double mutant (Figure S4). In summary, restored induction of *LHCB2.4*, *GLK1* and *GLK2* expression could explain the wild type chloroplast morphology in *papp5crd* during the early light response.

### Significant tetrapyrrole accumulation observed also in *papp5crd*

**Figure 5:**
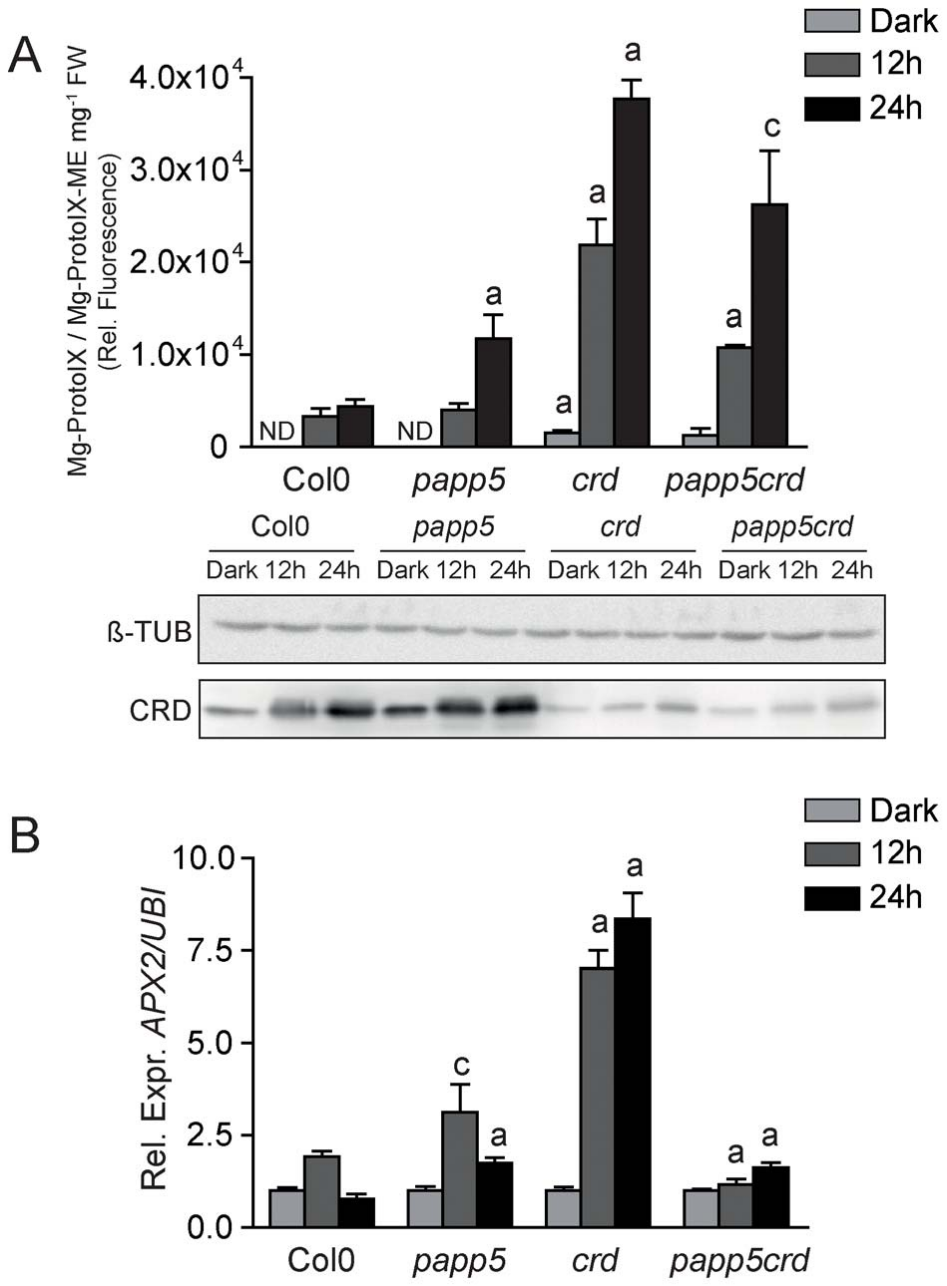
Tetrapyrrole accumulation maintained in *papp5crd*. A) Relative fluorescence corresponding to Mg-ProtoIX and Mg-ProtoIX-ME in seedlings from Col0, *papp5*, *crd*, and *papp5crd* during the dark to light transition. 3-d-old seedlings were transferred to constant light (100 µmol photons light cm^−2^ sec^−1^) and samples were collected following 12 h and 24 h exposure. Each data point represents the mean (± SD) of four independent biological replicates. Fluorescence data is complemented with Western blot analysis of the CRD protein levels in the different genotypes. β-Tubulin was used as protein loading control. B) Relative expression of *APX2* (At3g09640) in Col0, *papp5*, *crd*, and *papp5crd*. Expression levels were compared to the respective dark control for each genotype and relative expression was calculated using Ubiquitin-protein ligase (At4g36800) as a reference gene. Each bar represents the mean (± SD) of at least three independent biological samples. Significant differences relative to Col0 (*crd* and *papp5*) and to *crd* (*papp5crd*) were calculated according to *t-test* (a, P<0.001; b, c, P<0.01)

Due to impaired Mg-ProtoIX-ME cyclase activity in the *crd* mutant, the *crd* plants accumulate large pools of tetrapyrrole intermediates and the accumulation has been shown to correlate with a negative effect on the *PhANG* expression [28]. The accumulation of Mg-ProtoIX and its methyl ester, Mg-ProtoIX-ME was determined in the different genotypes during the first 24 hours of illumination. In wild type, Mg-ProtoIX/Mg-ProtoIX-ME accumulated slightly in the light samples compared to the dark (Figure 5A). In *papp5*, the accumulation of tetrapyrroles was slightly higher compared to wild type which also fits with the faster accumulation of chlorophyll in response to light in *papp5* (Figure 3). However, in *crd* and *papp5crd* a massive accumulation Mg-ProtoIX/Mg-ProtoIX-ME was observed (Figure 5A). Although the accumulation of tetrapyrroles was lower in *papp5crd* compared to *crd*, the *papp5* mutation does not rescue the cyclase activity or restores the amount of CRD protein (Figure 5A).

Tetrapyrroles are photoreactive molecules and in response to light, ROS could be generated [38]. Induction *ASCORBATE PEROXIDASE 2* (*APX2*) expression is one of the most commonly used markers for the H_2_O_2_ response and expression of *APX2* was therefore investigated in the different genotypes following exposure to light for 12 and 24 hours. In wild type and *papp5*, *APX2* expression was slightly induced following 12 hours light exposure but following 24 hours illumination the expression levels dropped to control levels (Figure 5B). After 12 hours light exposure the chloroplasts are still immature (Figure 2) and most likely not able to completely quench the light. However, after 24 hours in the light the chloroplasts appear mature and the different photosynthetic complexes are able to efficiently absorb the light and as a consequence less ROS is produced (Figure 5B). The *crd* seedlings displayed much stronger induction of *APX2* expression compared to wild type and *papp5*. Furthermore, the *APX2* expression level did not drop following 24 hours illumination in the *crd* mutant. This coincides with the undeveloped chloroplasts observed in the *crd* mutant at this time point (Figure 2). In contrast to the *crd* single mutant, the *papp5crd* double mutant did not show elevated *APX2* expression (Figure 5B). This is despite the fact that the *papp5crd* double mutant maintains very high pools of accumulated tetrapyrroles (Figure 5A). Thus, the ROS mediated signal triggering *APX2* induction is not correlated with the accumulated pools of tetrapyrroles but rather to the developmental stage of the chloroplasts.

### PAPP5 is a component of a cytosolic protein complex that binds Mg-ProtoIX

**Figure 6:**
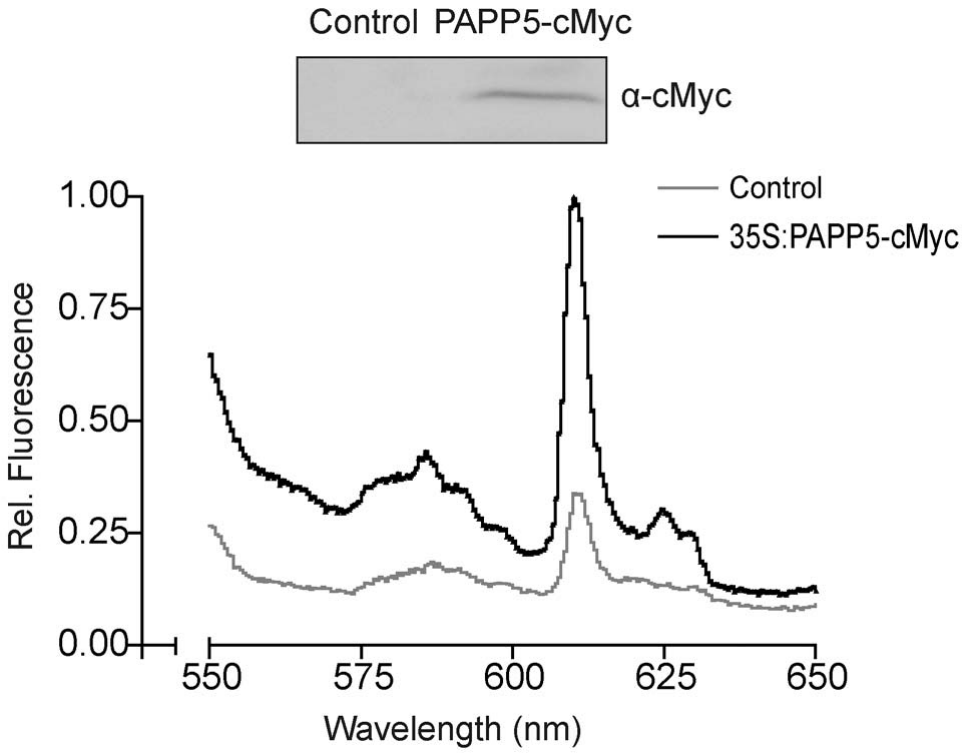
*In planta* tetrapyrrole binding assay. Tetrapyrrole fluorescence spectra from CoIP assay using pGWB16-empty (control) and 35-PAPP5-cMyc (pGWB16) transformed tobacco plants. The native PAPP5 protein complex was immunoprecipitated from PAPP5 overexpressing tobacco plants with anti c-myc antibody and incubated with Mg-ProtoIX solution. The amount of tetrapyrrole bound to the protein complex was estimated by fluorescence. The excitation wavelength used was 416 nm. The Western blot of the samples is shown in the upper panel using the anti-myc antibody.

In contrast to the *crd* single mutant, *papp5crd* demonstrated wild type expression of *PhANGs* and normal development of chloroplast in response to light (Figure 2–4) despite significant accumulation of the chlorophyll intermediates Mg-ProtoIX/Mg-ProtoIX-ME. Thus, the results suggest that PAPP5 is involved in the perception and/or the mediation of the tetrapyrrole triggered retrograde signal. To determine if there is a direct interaction between PAPP5 and Mg-ProtoIX, recombinant full-length PAPP5 and TPR-PAPP5, lacking the TPR domain, was expressed and Mg-ProtoIX bound by the proteins was quantified by spectrofluorometry (Figure S5A). Using this method, no direct interaction between Mg-ProtoIX and PAPP5 could be detected. However, we also tested *in vivo* if PAPP5 is a component of a distinct protein complex that is able to bind Mg-ProtoIX. We transiently expressed PAPP5 fused to a cMyc-tag in tobacco plants, immunoprecipitated PAPP5 containing protein complexes and incubated the isolated complexes with Mg-ProtoIX as it was done for the recombinant proteins (Figure S5A). The experimental procedure used in this assay requires elution of the protein complex with acidic buffer in order to break the Immunoglobulin-to-antigen binding. Under these acidic conditions, the Mg-ProtoIX molecule is unstable and the Mg^2+^ ion is released from the tetrapyrrole ring (Figure S5B) and as a consequence we detected ProtoIX instead of Mg-ProtoIX. It is clear from this *in vivo* approach that protein(s) immunoprecipitated with PAPP5 can bind Mg-ProtoIX (Figure 6). Possibly another protein partner(s) is required for PAPP5 to be able to bind the tetrapyrrole or PAPP5 is associated with another tetrapyrrole binding protein in a complex.

### PAPP5 responds to the tetrapyrrole mediated plastid signal and acts as a negative regulator of *PhANG* expression

**Figure 7:**
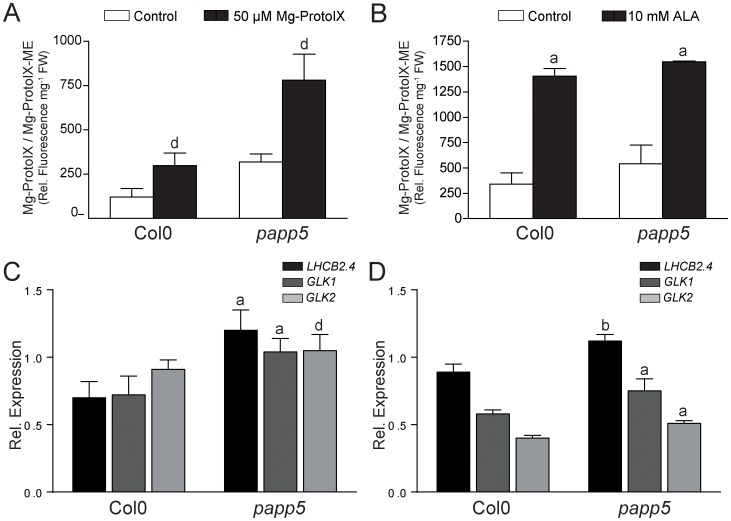
The *papp5* mutant is insensitive to tetrapyrrole feeding. Feeding experiments were performed to increase the levels of tetrapyrroles in the plants. During the dark period, two-week-old plants from Col0 and *papp5* grown under LD conditions (15 hours light/9 hours dark) were treated with A) 50 µM Mg-ProtoIX or B) 10 mM ALA. Relative fluorescence corresponding to Mg-ProtoIX and Mg-ProtoIX-ME is shown. Samples were collected 4 h and 1 h into the light period following the Mg-ProtoIX and ALA treatment, respectively. Each bar represents the mean (± SD) of three independent biological replicates. Relative expression of *LHCB2.4* (At3g27690), *GLK1* (At2g20570) and *GLK2* (At5g44190) in Col0 and *papp5* plants following C) Mg-ProtoIX or D) ALA feeding is shown. Expression levels were compared to the respective mock control for each genotype and relative expression was calculated using Ubiquitin-like protein (At4g36800) as internal standard. Each bar represents the mean (± SD) of at least three independent biological replicates. Significant differences relative to untreated control or Col0 were calculated according to *t-test* (a, P<0.001; b, P<0.005; d, P<0.05).

In order to test the link between PAPP5 and the tetrapyrrole-mediated retrograde signalling pathway we analysed *PhANG* expression in wild type and *papp5* following feeding with Mg-ProtoIX or 5-aminolevulinic acid (ALA). ALA is the first metabolite in the tetrapyrrole biosynthetic pathway that leads to chlorophyll. Mg-ProtoIX feeding results in increased tetrapyrrole levels in the cytosol whereas ALA treatment increases tetrapyrrole content in the chloroplast which better represents a biological situation when flux through the tetrapyrrole pathway is altered. Mg-ProtoIX (Figure 7A) and ALA (Figure 7B) feeding resulted in a significant accumulation of Mg-ProtoIX both in wild type and *papp5* compared to the mock samples. Following the Mg-ProtoIX and ALA feeding expression of *LHCB2.4*, *GLK1* and *GLK2* was repressed in wild type (Figure 7C–D). This is in agreement with several published reports on the negative effects of tetrapyrroles on *PhANG* expression [15], [16], [17], [18], [28]. However, in the *papp5* mutant, *LHCB2.4*, *GLK1* and *GLK2* expression was insensitive to Mg-ProtoIX feeding and no repression in expression levels could be detected (Figure 7C). Following ALA feeding repression of *GLK1* and *GLK2* was observed in the *papp5* mutant but the repression was significantly less compared to wild type (Figure 7D). Thus, these results suggest that PAPP5 is required to respond correctly to accumulation of tetrapyrroles in the cell.

### Phosphatase activity is required to mediate the tetrapyrrole related plastid signal to the nucleus

**Figure 8:**
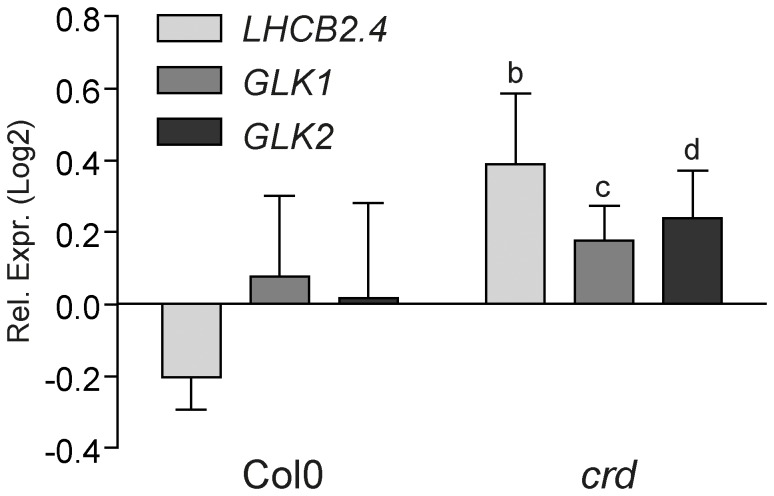
Okadaic acid treatment phenocopies *papp5crd* in *crd* single mutant. Relative expression of *LHCB2.4* (At3g27690), *GLK1* (At2g20570) and *GLK2* (At5g44190) in Col0 and *crd* plants following treatment with 10 nM okadaic acid during de-etiolation. Samples were collected following 12 h exposure to light. Each bar represents the mean (± SD) of three independent biological replicates. Significant differences relative to Col0 were calculated according to *t-test* (b, P<0.005; c, P<0.01; d, P<0.05).

To address whether PAPP5 phosphatase activity is required to transmit the tetrapyrrole-mediated plastid signal to the nucleus, we used okadaic acid to inhibit the phosphatase activity during the first 12 hours of illumination. Okadaic acid is a cytotoxin derived from algae that specifically blocks the PP5-2A type phosphatases [39] and it has been shown to reduce PAPP5 activity *in vitro* [40]. *LHCB2.4*, *GLK1* and *GLK2* expression was significantly induced following okadaic acid treatment in *crd* compared to the untreated control (Figure 8). Thus, blocking phosphatase activity phenocopied the *papp5crd* phenotype in the *crd* single mutant and reverts the suppression of *PhANG* expression. These data demonstrates that phosphatase activity is important to mediate the retrograde signal to regulate *PhANG* expression in *crd*.

## Discussion

**Figure 9:**
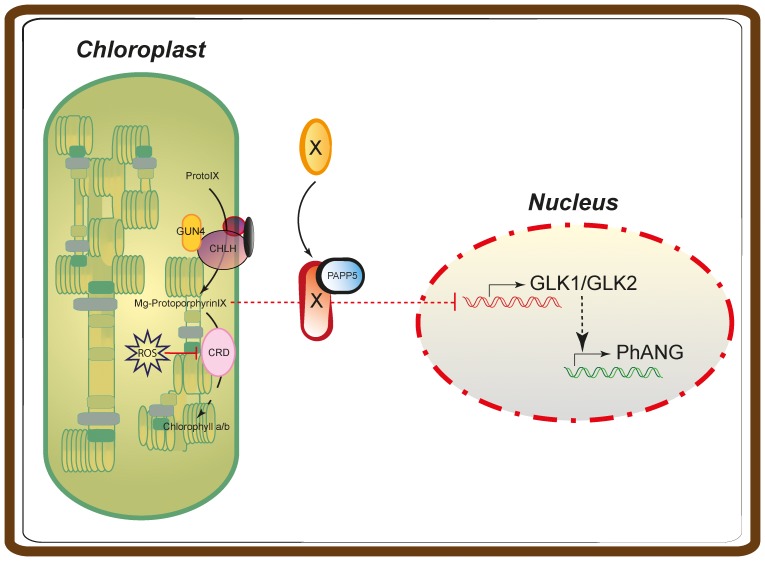
Working model for the role of PAPP5 in tetrapyrrole mediated plastid signalling. PAPP5 pereceives an inbalance in the tetrapyrrole biosynthesis through the accumulation of Mg-ProtoIX/Mg-ProtoIXME and acts as a negative regulator of chloroplast biogenesis and development. The tetrapyrrole/PAPP5-mediated plastid signal blocks the induction of the genes encoding the GLK1/2 transcription factors. GLK1 and GLK2 are essential for the induction of *PhANG* expression and chloroplast development.

The regulation of *PhANG* expression is very complex and involves signals from multiple signalling pathways, such as those triggered by light, circadian clock and signals originating in the plastids [41]. Although light and plastid signals trigger distinct signalling pathways [7], it has been shown that plastid signals and light signals can regulate *PhANG* expression using common or adjacent promoter elements [16], [42]. Plastid signals have been suggested to play an essential role in the regulation of *PhANG* expression when proplastids develop into chloroplasts [7], [41], [43]. One of the plastid signals described to regulate the expression of *PhANGs* is linked to the tetrapyrrole biosynthesis and we have demonstrated that PAPP5 phosphatase activity is required to transmit the tetrapyrrole-mediated plastid signal to the nucleus during chloroplast development. Our results further suggest that *PhANG* expression is controlled by a balance between inductive light signalling pathways and a repressive plastid signal triggered by impaired flux through the chlorophyll biosynthesis (Figure 9).

In the double mutant *papp5crd*, the *crd* phenotype is restored and in contrast to the *crd* single mutant, *papp5crd* accumulated wild type levels of chlorophyll, developed proper chloroplasts and showed normal induction of *PhANG* expression in response to light (Figures 1–4). The recovery of the pale phenotype is observed in *papp5crd* even though *papp5crd* showed a massive accumulation Mg-ProtoIX/Mg-ProtoIX-ME in the light, similar to what was shown for *crd* (Figure 5A). Furthermore, the *papp5* mutation does not rescue the cyclase activity in the *crd* background or restores the amount of CRD protein (Figure 5A). Thus, PAPP5 is most likely a component in the plastid signalling pathway down stream of the tetrapyrrole Mg-ProtoIX/Mg-ProtoIX-ME. In support of this, the *papp5* single mutant demonstrated higher levels of chlorophyll following both 12 and 24 hours light exposure compared to wild type suggesting that PAPP5 acts as a negative regulator of chlorophyll biosynthesis during the early light response. In addition, *PhANG* transcript levels following 12 and 24 hours light exposure were significantly higher in *papp5* compared to wild type (Figure S4). Thus, our result suggests that PAPP5 receives an inbalance in the tetrapyrrole biosynthesis through the accumulation of Mg-ProtoIX and acts as a repressor during chloroplast biogenesis and development.

The deduced amino acid sequence of PAPP5 has two distinctive domains, the N-terminal domain containing the three tetratricopeptide repeats (TPRs) responsible for the protein-protein interaction and the C-terminal domain containing the highly conserved signature motifs of a type 2A serine/threonine protein phosphatase (PP2Ac). This domain structure is a characteristic feature of members of the type 5 serine/threonine protein phosphatase (PP5) subfamily [44]. PAPP5 phosphatase activity was shown to be essential to mediate the retrograde signal and to suppress *PhANG* expression in the *crd* mutant (Figure 8). Inhibition of phosphatase activity by okadaic acid phenocopied the *papp5crd* phenotype in the *crd* single mutant and reverted the suppression of *PhANG* expression in *crd* (Figure 8). Furthermore, a direct link between the tetrapyrrole Mg-ProtoIX and PAPP5 was demonstrated in a co-immunoprecipitation assay performed in tobacco leaves where PAPP5 was shown to interact with protein(s) that binds Mg-ProtoIX (Figure 6). Thus, *in vivo* PAPP5 appears to be a component of a distinct protein complex that is able to bind Mg-ProtoIX. Possibly another protein partner(s) is required for PAPP5 to be able to bind the tetrapyrrole or PAPP5 is associated with another tetrapyrrole binding protein in a complex. Besides the interaction with PHYA/PHYB [24], PAPP5 has been demonstrated to interact *in vivo* with HSP90, PP2A and ASK1 [40], [45], [46], [47]. HSP90 was recently shown to be required for the Mg-ProtoIX/GUN5 mediated plastid signal and the interaction between HSP90 and Mg-ProtoIX was shown to inhibit ATP-ase activity of HSP90 [17]. Possibly, HSP90 and PAPP5 are components in the same complex responding to and mediating the tetrapyrrole signal from the plastids.

Feeding experiments with Mg-ProtoIX or 5-aminolevulinic acid (ALA) further demonstrated a connection between PAPP5 and the tetrapyrroles. Feeding resulted in a significant accumulation of Mg-ProtoIX both in wild type and *papp5* compared to the mock samples and expression of *PhANGs* was repressed in wild type (Figure 7C–D). In contrast, *LHCB2.4*, *GLK1* and *GLK2* expression was insensitive to Mg-ProtoIX feeding and no repression of the *PhANGs* could be detected in the *papp5* mutant. Following ALA feeding the repression of *GLK1* and *GLK2* was observed but was significantly less compared to wild type (Figure 7C–D). To act as signalling molecule and to affect the activity of PAPP5 and later the expression of the *PhANGs*, the chlorophyll intermediate must reach the cytosol. Numerous porphyrins synthesized in the chloroplast, e.g. chlorophyll catabolites, heme and heme precursors have been found to exit the chloroplast [36], [48], [49], [50]. Mg-ProtoIX/Mg-ProtoIX-ME has also been shown to accumulate in the cytosol during stress conditions and it was proposed that the tetrapyrroles are transported across the membrane acting as putative signalling metabolites [15], [36]. However, the route(s) for transport of any tetrapyrroles from the chloroplast or the components involved in the transport are still unknown. It has also been suggested that ROS accumulation is in fact the origin of the tetrapyrrole mediated plastid signal instead of the specific accumulation of Mg-ProtoIX/Mg-ProtoIX-ME [22], [23]. The different ROS species activate distinct signalling pathways and the release of ROS could be an alternative explanation for the role of tetrapyrrole intermediates in retrograde signalling because many porphyrins are photoreactive and generate ROS in the presence of light [38]. Under our experimental conditions, *papp5crd* accumulated high levels of Mg-ProtoIX/Mg-ProtoIX-ME but in contrast to the *crd* single mutant where *APX2* expression was strongly induced, no induction of *APX2* expression was detected in *papp5crd* (Figure 5). Thus, despite the tetrapyrrole accumulation, *PhANG* expression was restored and *APX2* expression was similar in *papp5crd* to what was observed in wild type. Possibly PAPP5 could be required to fully induce *APX2* expression in response to ROS or more likely, the ROS levels are correlated with the degree of chloroplast development in *crd* and *papp5crd* (Figure 2). *APX2* expression was induced following 12 hours light exposure in wild type and the *papp5* mutant (Figure 5). However, the expression levels were reduced after 24 hours light exposure that correlates with the time point when functional chloroplasts have developed in wild type and *papp5* (Fig. 2 and 5). *APX2* expression level did not drop following 24 hours illumination in the *crd* mutant which coincides with the undeveloped chloroplasts observed in the *crd* mutant at this time point (Figure 2). Thus, the ROS triggered *APX2* induction does not appear to be correlated with the accumulated pools of tetrapyrroles but rather to the functional stage of the chloroplasts. Furthermore, specific ROS eliminators were shown to only partly reverse the tetrapyrrole-triggered repression of *LHCB* [15] and expression of marker genes for ROS were not different in *gun5* mutant, impaired in the tetrapyrrole-mediated pathway, compared to wild type [51]. Taken together, the tetrapyrrole mediated signal is most likely not related to an altered accumulation of ROS.

Our results suggest that expression of *GLK1* and *GLK2* is repressed by the tetrapyrrole-PAPP5 mediated plastid signal during the early light response (Figure 4, 9). Higher expression levels of *GLK1* and *GLK2* were observed in *papp5* compared to wild type whereas in *crd* the induction of *GLK1/2* following light exposure was significantly suppressed (Figure 4B–C). The *papp5crd* seedlings on the other hand displayed *GLK1* and *GLK2* expression similar to wild type (Figure 4B–C). GLK1 and GLK2 are transcription factors shown to be required for chlorophyll biosynthesis and photosynthesis related gene expression during chloroplast development [25], [37]. Our results suggest that tetrapyrrole accumulation generates a PAPP5-mediated plastid signal involving a cytosolic protein that suppresses *GLK1/2* expression. The two *GLK* genes have been shown previously to respond to plastid retrograde signals and expression of *GLK1/2* was shown to be sensitive to the feedback signalling from the chloroplast suggesting that they may operate downstream of plastid retrograde signalling [25]. Furthermore, the *crd* and *glk1glk2* mutants share many common features, similarly to *crd*, *glk1glk2* is pale and contain thylakoid membranes without proper grana. In addition, the *glk1glk2* mutant exhibits reduced transcript and protein levels for nuclear-encoded photosynthetic genes, especially those associated with chlorophyll biosynthesis and light harvesting [28], [52]. This suggests that the phenotype observed in the *crd* mutant could partly be explained by the mis-regulation of the GLK transcription factors. The recovery of the *crd* phenotype in *papp5crd* would then be established through higher levels of chlorophyll binding proteins in the chloroplast and improved photosynthetic performance. In summary, our results demonstrate that the master regulators of *PhANG* expression and chloroplast development, the GLKs, are regulated by signals originating in the plastids communicating an imbalance in the biosynthesis of the photosynthetic pigments. This signal is transmitted by an unknown factor probably activated upon PAPP5-mediated dephosphorylation. This factor acts as a negative regulator of *GLK* expression and consequently also of chloroplast biogenesis (Figure 9). Thus, chloroplast development is controlled by a delicate interplay between light and plastid signalling pathways.

## Supporting Information

Figure S1 **Characterization of** ***papp5-1*** **mutant allele.** A) Position of T-DNA insertion in the *papp5-1* allele in the At2g42810 gene and B) quantitative RT-PCR of *PAPP5* transcripts in homozygous *papp5-1* seedlings compared to wild type.
(PDF)

Figure S2 **Characterization of** ***phyB, gun5, phyBcrd*** **and** ***papp5gun5*** **plants.** 6-week-old plants of wild type, *crd*, *phyB* and *phyBcrd* grown on soil under short day conditions (9 hours light/15 hours dark). A) Representative images from 6-week-old plants. Scale bar = 1 cm. B) Hypocotyl length in seedlings grown in constant red light (630 nm at 20 µmol cm^−2^ s^−1^) for 5 days. The data is presented as mean (± SD) where *n* = 100 seedlings. C) Representative images from 6-week-old plants of wild type, *gun5* and *papp5gun5* grown in the same condition as described above. Chlorophyll data represents the mean (± SD) of three independent biological replicates. Significant differences relative to Col0 were calculated according to *t-test* (d, P≤0.05).
(PDF)

Figure S3 **Flowering time in** ***crd*** **and** ***papp5crd*** **plants.** Flowering time was determined in Col0, *papp5*, *crd* and *papp5crd Arabidopsis thaliana* plants grown in SD by counting A) the number of leaves when floral buds were visible at the centre of the rosette and B) the number of days from sowing to the day when floral buds appear. The results are presented as mean (± SD) where *n* = 12–15 plants. Significant differences relative to Col0 (*crd*) and to *crd* (*papp5crd*) were calculated according to *t-test* (a, P≤0.001).
(PDF)

Figure S4 ***PhANG*** **and** ***APX2*** **expression during chloroplast development.** Relative expression levels of A) *LHCB2.4* (At3g27690), B) *GLK1* (At2g20570), C) *GLK2* (At5g44190) and D) *APX2* (At3g09640) in seedlings grown for three days in dark and exposed to 12 h and 24 h of illumination. Expression levels were compared to the Col0 level at each time point and relative expression was calculated using Ubiquitin-protein ligase (At4g36800) as a reference gene. E) Expression levels of *LHCB2.4, GLK1* and *GLK2* in *papp5crd* compared to *crd*. Data represents the mean (± SD) from three independent biological replicates.
(PDF)

Figure S5 **Test for direct interaction between Mg-ProtoIX and PAPP5** ***in vitro*** **.** A) LacZ was used as a control protein, PAPP5 and TPR-PAPP5 (PAPP5 lacking the TPR domain) proteins were expressed and purified. Target-6xHis proteins were mixed with the indicated concentrations of Mg-ProtoIX and then isolated with Ni-agarose beads. After elution from the beads with imidazole, protein-bound Mg-ProtoIX was quantified using spectrofluorometry. Mg-ProtoIX fluorescence intensity was then normalized to the eluted proteins quantified by immunoblot analysis with antibodies against 6xHis (upper panel). Data is expressed as a mean (± SD) from three independent samples. B) Normalized spectra corresponding to Mg-ProtoIX dissolved in basic solution and in the acidic solution used in for the elution in the *in vivo* experiment (presented in Figure 6).
(PDF)

Table S1 **Primers sequences used for the experiments presented.**
(PDF)
