# Supplementary figures and images for: PAPP5 Is Involved in the Tetrapyrrole Mediated Plastid Signalling during Chloroplast Development
Source: PLoS One. 2013 Mar 29;8(3):e60305. doi: 10.1371/journal.pone.0060305 (PMC3612061; doi:10.1371/journal.pone.0060305)

# Supplemental Figure1

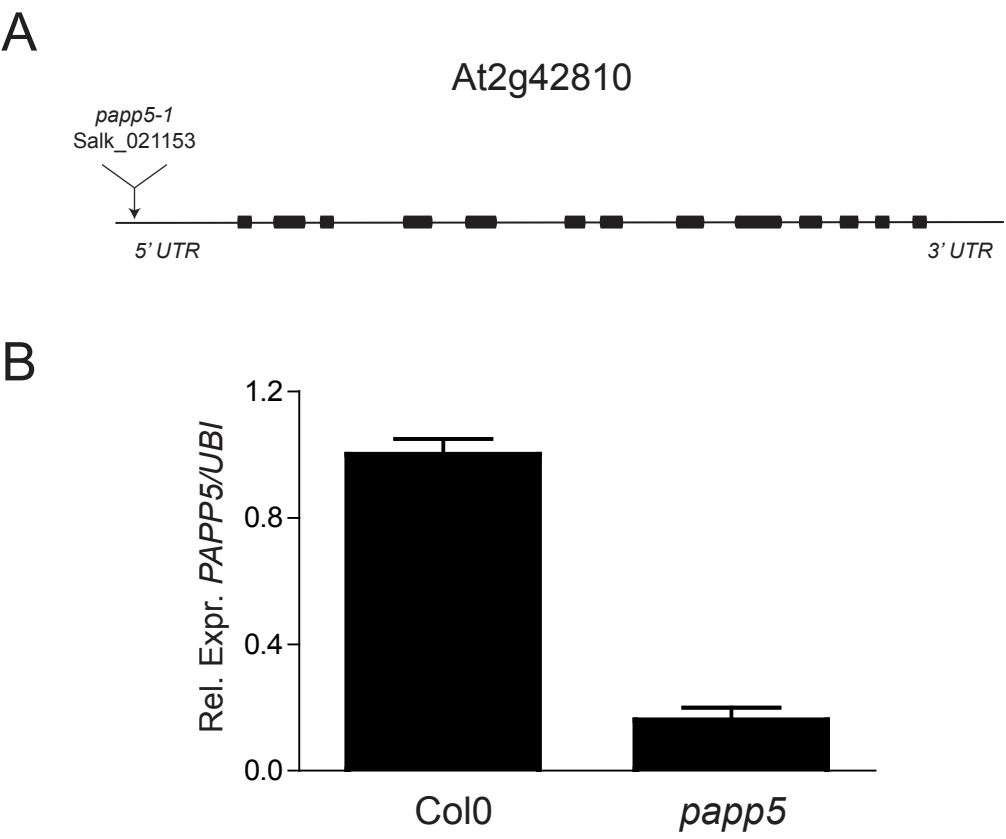

Supplement: Figure S1 — Characterization of papp5-1 mutant allele. A) Position of T-DNA insertion in the papp5-1 allele in the At2g42810 gene and B) quantitative RT-PCR of PAPP5 transcripts in homozygous papp5-1 seedlings compared to wild type. (PDF) [file pone.0060305.s001.pdf]

# Supplemental Figure2

A

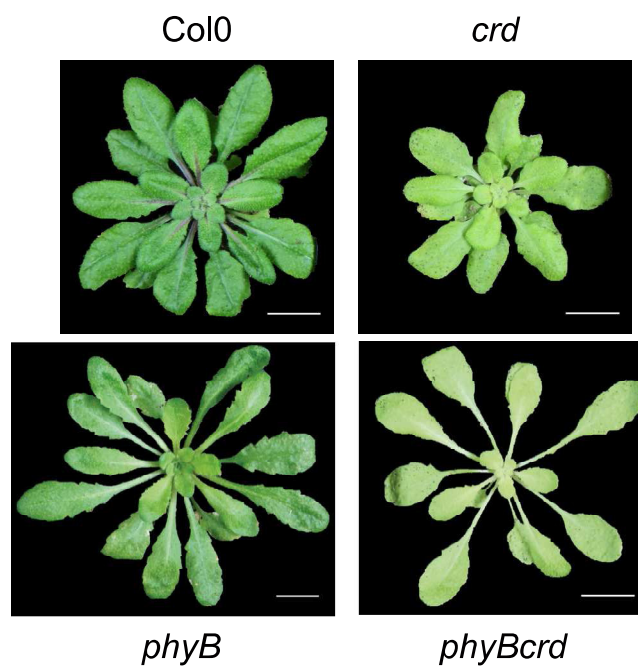

B

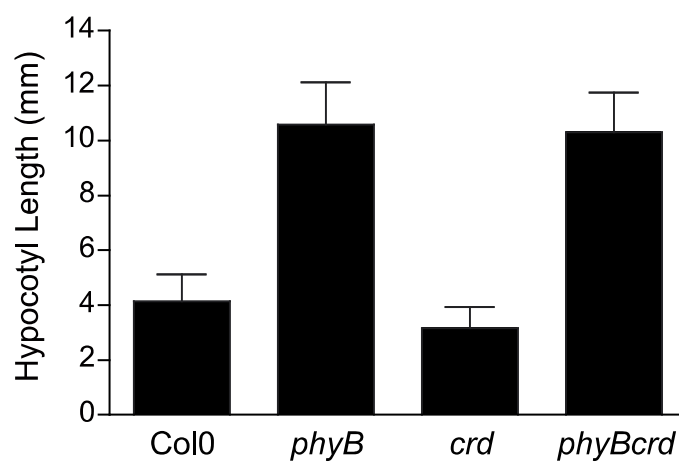

C

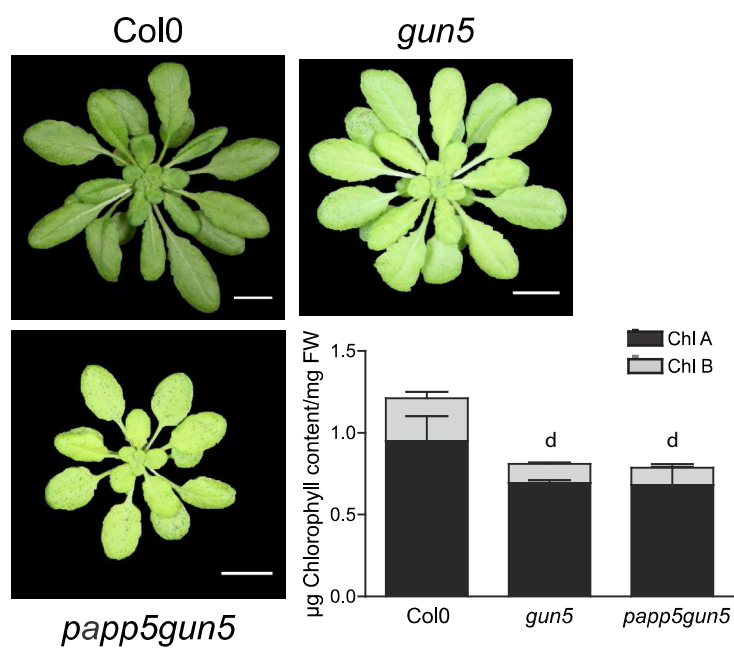

Supplement: Figure S2 — Characterization of phyB, gun5, phyBcrd and papp5gun5 plants. 6-week-old plants of wild type, crd, phyB and phyBcrd grown on soil under short day conditions (9 hours light/15 hours dark). A) Representative images from 6-week-old plants. Scale bar = 1 cm. B) Hypocotyl length in seedlings grown in constant red light (630 nm at 20 µmol cm−2 s−1) for 5 days. The data is presented as mean (± SD) where n = 100 seedlings. C) Representative images from 6-week-old plants of wild type, gun5 and papp5gun5 grown in the same condition as described above. Chlorophyll data represents the mean (± SD) of three independent biological replicates. Significant differences relative to Col0 were calculated according to t-test (d, P≤0.05). (PDF) [file pone.0060305.s002.pdf]

# Supplemental Figure3

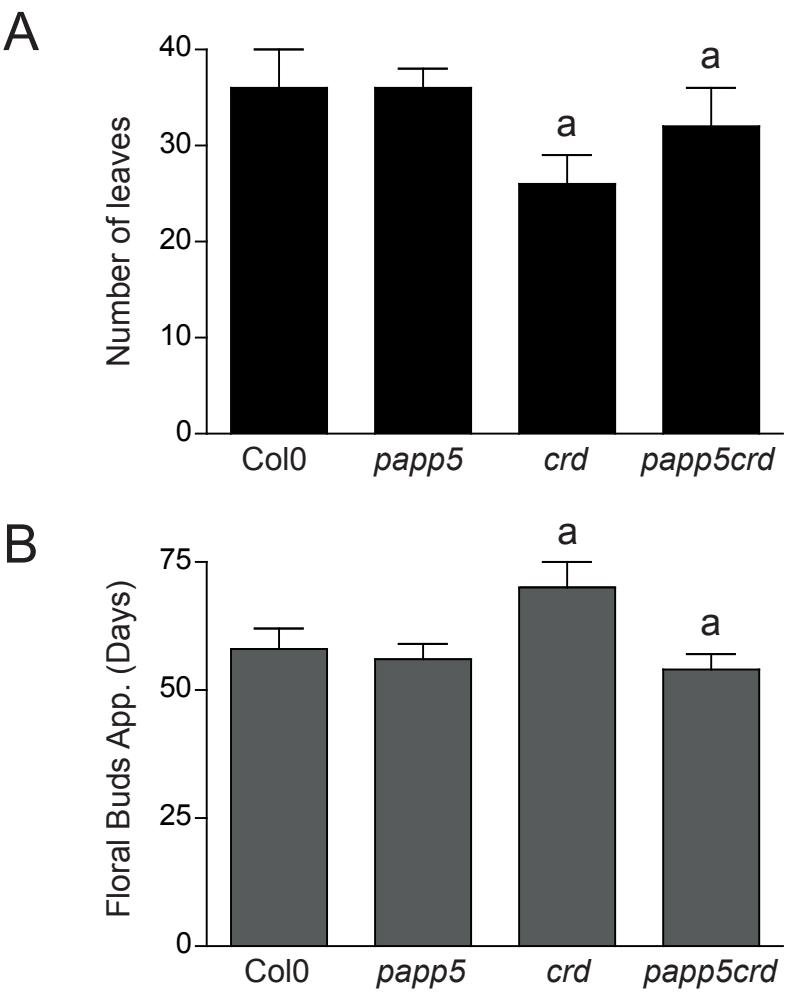

Supplement: Figure S3 — Flowering time in crd and papp5crd plants. Flowering time was determined in Col0, papp5, crd and papp5crd Arabidopsis thaliana plants grown in SD by counting A) the number of leaves when floral buds were visible at the centre of the rosette and B) the number of days from sowing to the day when floral buds appear. The results are presented as mean (± SD) where n = 12–15 plants. Significant differences relative to Col0 (crd) and to crd (papp5crd) were calculated according to t-test (a, P≤0.001). (PDF) [file pone.0060305.s003.pdf]

# Supplemental Figure4

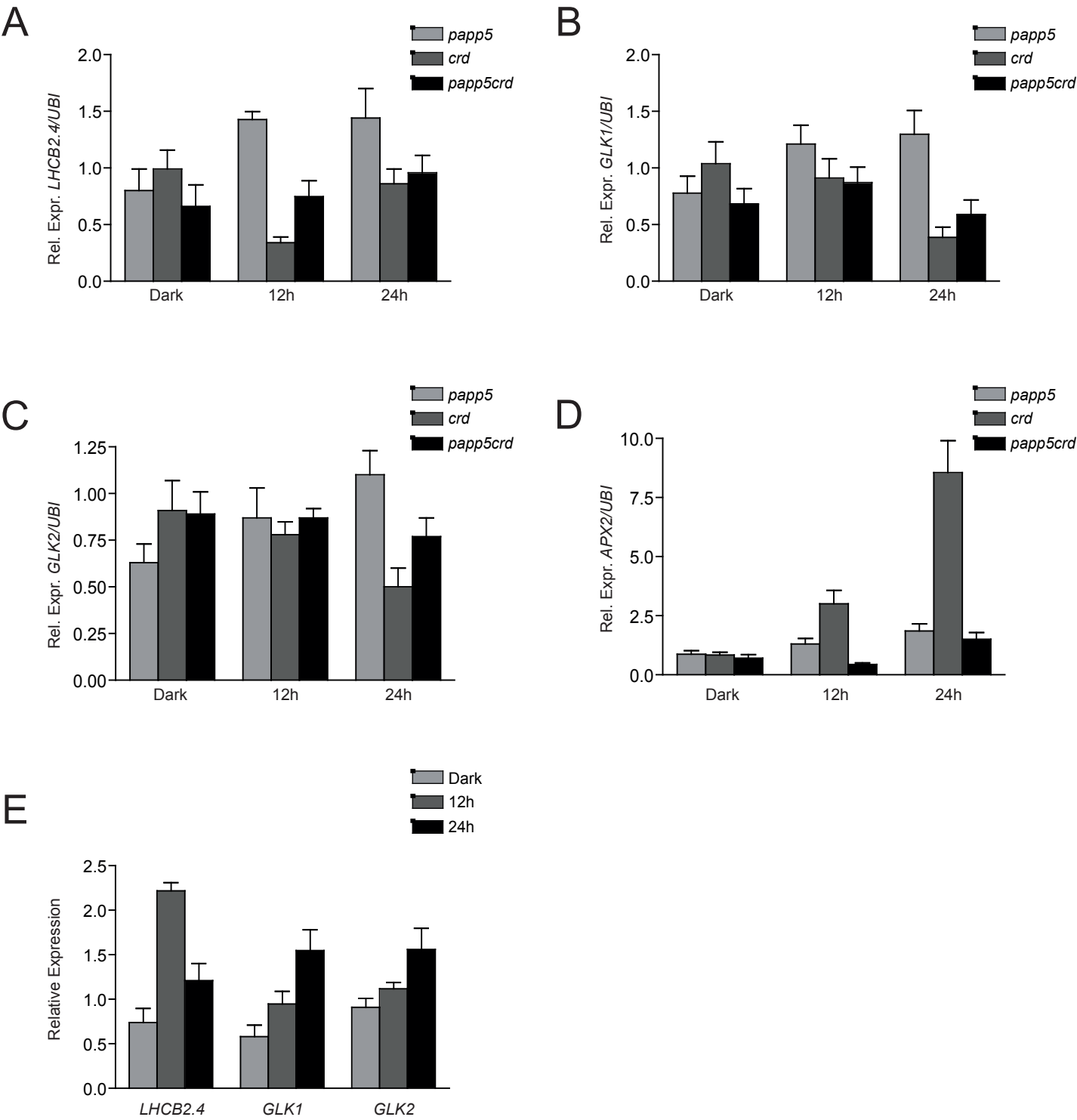

Supplement: Figure S4 — PhANG and APX2 expression during chloroplast development. Relative expression levels of A) LHCB2.4 (At3g27690), B) GLK1 (At2g20570), C) GLK2 (At5g44190) and D) APX2 (At3g09640) in seedlings grown for three days in dark and exposed to 12 h and 24 h of illumination. Expression levels were compared to the Col0 level at each time point and relative expression was calculated using Ubiquitin-protein ligase (At4g36800) as a reference gene. E) Expression levels of LHCB2.4, GLK1 and GLK2 in papp5crd compared to crd. Data represents the mean (± SD) from three independent biological replicates. (PDF) [file pone.0060305.s004.pdf]

# Supplemental Figure5

A

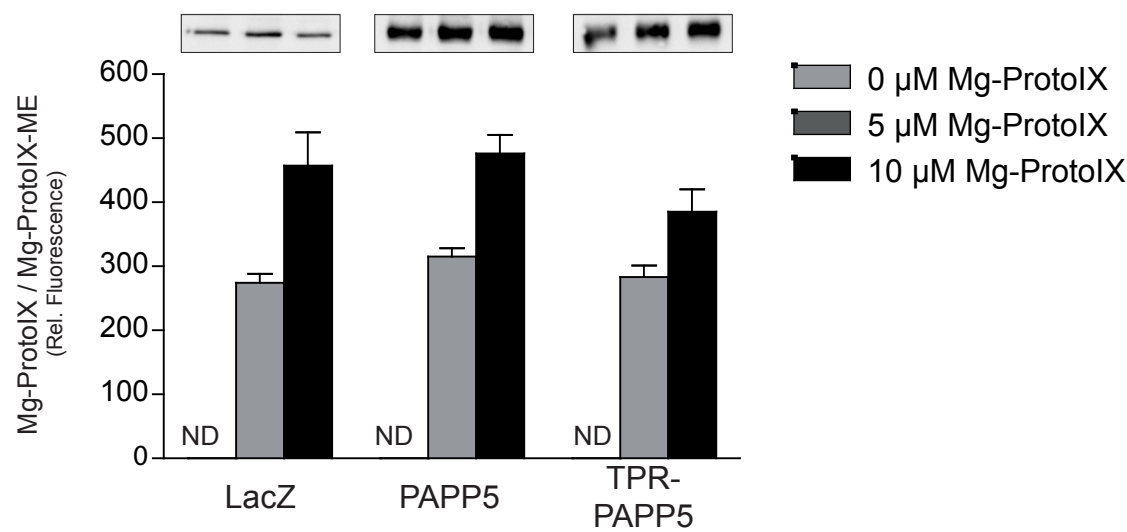

B

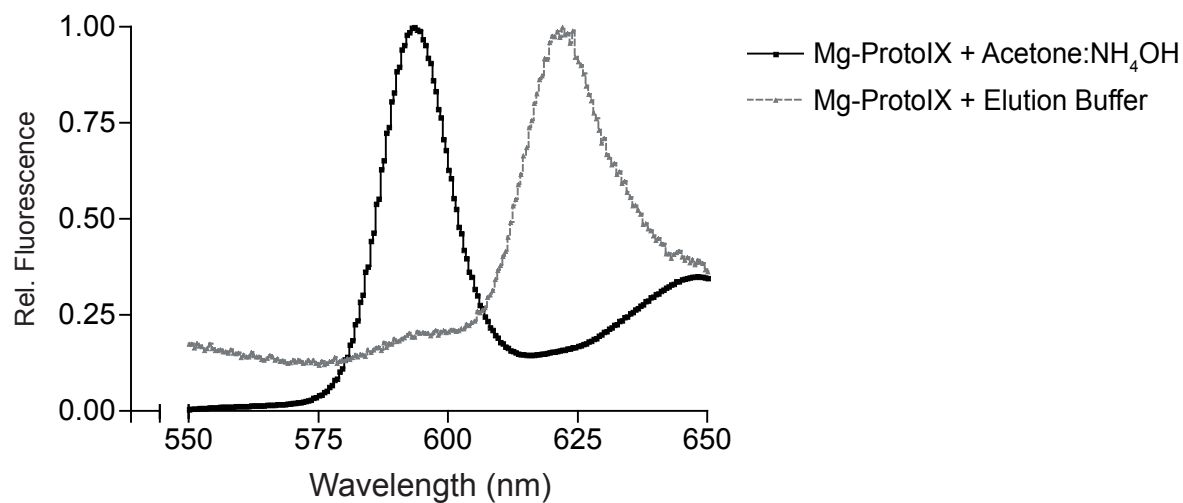

Supplement: Figure S5 — Test for direct interaction between Mg-ProtoIX and PAPP5 in vitro . A) LacZ was used as a control protein, PAPP5 and TPR-PAPP5 (PAPP5 lacking the TPR domain) proteins were expressed and purified. Target-6xHis proteins were mixed with the indicated concentrations of Mg-ProtoIX and then isolated with Ni-agarose beads. After elution from the beads with imidazole, protein-bound Mg-ProtoIX was quantified using spectrofluorometry. Mg-ProtoIX fluorescence intensity was then normalized to the eluted proteins quantified by immunoblot analysis with antibodies against 6xHis (upper panel). Data is expressed as a mean (± SD) from three independent samples. B) Normalized spectra corresponding to Mg-ProtoIX dissolved in basic solution and in the acidic solution used in for the elution in the in vivo experiment (presented in Figure 6). (PDF) [file pone.0060305.s005.pdf]
