# Supplementary material for: PAPP5 Is Involved in the Tetrapyrrole Mediated Plastid Signalling during Chloroplast Development
Source: PLoS One. 2013 Mar 29;8(3):e60305. doi: 10.1371/journal.pone.0060305 (PMC3612061; doi:10.1371/journal.pone.0060305)
Supplement: Table S1 — Primers sequences used for the experiments presented. (PDF) [file pone.0060305.s006.pdf]

Table S1

Primers used for Q-rt-PCR

Ubiquitin-protein ligase-like protein, At4g36800	
UBI Fw	CTGTTCACGGAACCCAATTC
UBI Rev	GGAAAAAGGTCTGACCGACA
Chlorophyll *a/b* binding protein, Lhcb2.4, At3g27690	
Lhcb2.4 Fw	GCCATCCAACGATCTCCTC
Lhcb 2.4 Rev	TGGTCCGTACCAGATGCTC
GOLDEN2-like factor1, At2g20570	
GLK1 Fw	TTGGGTCTCCGATTCTCCCTAT
GLK1 Rev	GCAACTGGCGGTGCTCTAAAT
GOLDEN2-like factor2, At5g44190	
GLK2 Fw	TATCCAATGCCGGCCATTGC
GLK2 Rev	ATGTCGATGGGAGGATTAGTGGGT
PAPP5, At2g42810	
PAPP5 Fw	CGCTGTGTATTGGGCAAATCGTG
PAPP5 Rev	ACCACGCCTGTAATAGCCCTTAG
APX2, At3g09640	
APX2-Fw	GGCTGGGACATTTGATGTG
APX2-Rev	AGGGAACAGCTCCTTGATAGG

Primers used for genotyping

CRD-RP	GATTTGGACCACGAATTTGTAACG
CRD-LP	TCTTTAAACTCCTTGTTCCTCACG
Papp5-RP	CAGCCACTGACTTTAGTCATGC
Papp5-LP	TTCAAGCTCGCGCTATATCAC
Lba1	TGGTTCACGTAGTGGGCCATCG

Primers used for recombinant protein cloning

papp5-TOPO-Fw	CACCATGGAGACCAAGAATGAGAATT
papp5-TOPO-Rev	TTAGTTGAACATCCTGAGAAAGTTG
papp5-TPR-His-F	caccatgCCACAATATTCTGGTGCTAG
